# Functional Analysis of Maize Silk-Specific *ZmbZIP25* Promoter

**DOI:** 10.3390/ijms19030822

**Published:** 2018-03-12

**Authors:** Wanying Li, Dan Yu, Jingjuan Yu, Dengyun Zhu, Qian Zhao

**Affiliations:** State Key Laboratory of Agrobiotechnology, College of Biological Sciences, China Agricultural University, No. 2 Yuanmingyuan West Road, Haidian District, Beijing 100193, China; liwanyingcau@foxmail.com (W.L.); danyupw155@yeah.net (D.Y.); yujj@cau.edu.cn (J.Y.); zhudy@cau.edu.cn (D.Z.)

**Keywords:** bZIP transcription factor, intron, maize, silk specificity, 5′-flanking sequence

## Abstract

ZmbZIP25 (*Zea mays* bZIP (basic leucine zipper) transcription factor 25) is a function-unknown protein that belongs to the D group of the bZIP transcription factor family. RNA-seq data showed that the expression of *ZmbZIP25* was tissue-specific in maize silks, and this specificity was confirmed by RT-PCR (reverse transcription-polymerase chain reaction). In situ RNA hybridization showed that *ZmbZIP25* was expressed exclusively in the xylem of maize silks. A 5′ RACE (rapid amplification of cDNA ends) assay identified an adenine residue as the transcription start site of the *ZmbZIP25* gene. To characterize this silk-specific promoter, we isolated and analyzed a 2450 bp (from −2083 to +367) and a 2600 bp sequence of *ZmbZIP25* (from −2083 to +517, the transcription start site was denoted +1). Stable expression assays in *Arabidopsis* showed that the expression of the reporter gene *GUS* driven by the 2450 bp *ZmbZIP25* 5′-flanking fragment occurred exclusively in the papillae of *Arabidopsis* stigmas. Furthermore, transient expression assays in maize indicated that *GUS* and *GFP* expression driven by the 2450 bp *ZmbZIP25* 5′-flanking sequences occurred only in maize silks and not in other tissues. However, no *GUS* or *GFP* expression was driven by the 2600 bp *ZmbZIP25* 5′-flanking sequences in either stable or transient expression assays. A series of deletion analyses of the 2450 bp *ZmbZIP25* 5′-flanking sequence was performed in transgenic *Arabidopsis* plants, and probable elements prediction analysis revealed the possible presence of negative regulatory elements within the 161 bp region from −1117 to −957 that were responsible for the specificity of the *ZmbZIP25* 5′-flanking sequence.

## 1. Introduction

Silk is a necessary part of the female reproductive organ in maize. Each maize silk is a specialized long trichome anchored to each ovule, which is functionally equivalent to the stigma and style in typical flowering plants [1,2,3]. Silk is responsible for catching pollen grains and supporting pollen hydration and germination. Pollen grains germinate to form a pollen tube after hydration on the silk; this tube grows parallel to the vascular bundles and reaches the ovule. The nutrition provided by pollen grains support the formation of 2-cm-long pollen tubes [1], while the subsequent nutrition and signal factors needed by the growing pollen tubes are provided by silk. The growth and development of maize silks directly relate to pollination and fertilization. Hairs located on the silk surface serve as receptive structures to aid pollen adhesion, hydration, and germination [4]. To accomplish these physiological processes, maize silks might express genes that are specific or at least preferential to silk.

Several genes that are specifically or preferentially expressed in stigmas have been identified. SSP (stigma-specific peroxidase), isolated from *Senecio squalidus* L., is expressed in specialized epidermal cells of stigmas. SSP may enhance protection against pathogen attack when the stigma is “primed” to receive pollen [5,6]. Other genes that function primarily in the stigma include the stigma-specific S locus receptor kinase from tobacco [7], proteinase inhibitors from *Nicotiana alata* stigmas [8], and genes involved in the self-incompatibility system of *Brassica* [9,10,11,12]. In maize, a transcriptome analysis showed that 1427 genes were specifically or preferentially expressed in silk [4]. Bioinformatic analyses revealed that many of these genes function in plant reproductive systems and that some of these genes encode amino acid transporters, peptide and oligopeptide transporters, and cysteine-rich receptor-like kinases [4]. However, few reports of research on silk-specific promoters exist. Tao et al. cloned a gene named *zmgrp5* (*Zea mays* glycine-rich protein 5), which encodes a 187-amino-acid glycine-rich protein that is expressed specifically in maize silks. Transient expression analyses revealed that this 1779 bp *zmgrp5* promoter fused with β-glucuronidase (*GUS*) was expressed in silk but not in leaf. Heterologous expression in *Arabidopsis* showed that a *zmgrp5* promoter-*GUS* fusion was expressed highly in stigmas and at low levels in the filaments and vascular elements of the petals [13].

Silks are one of the main ways in which fungi such as *Fusarium graminearum* invade maize [14]. Fungal spores grow and germinate on the silks, and hyphae then spread to the outside and inside of the silks until they infect the ovules. Silks are also susceptible to infection by insect pests such as corn earworm (*Helicoverpa zea*) [15]. The expression of some defense genes that benefit from silk-specific promoters in silk may increase resistance to fungi and pests. A *p1* (*pericarp1*) gene encoding a *Myb* transcription factor is driven by the putative silk-specific promoter *pSH64*, enhancing resistance to *Helicoverpa zea* [16]. Research on silk-specific promoters is also of great value in production. Sterility-related genes can be driven by silk-specific promoters to produce sterile female plants. Female sterility plays a role in genetic breeding and seed production by effectively preventing self-pollination. 

In this study, we showed that *ZmbZIP25* is expressed specifically in maize silk. Expression analysis of both maize and *Arabidopsis* showed that the region of *ZmbZIP25* from −2083 to +367 contains the promoter activity. A detailed promoter deletion analysis was performed and identified a region from −1117 to −957 that is required for *ZmbZIP25* specificity. Moreover, aberrant splicing of the second intron of *ZmbZIP25* might lead to the inability to express reporter genes in transgenic *Arabidopsis*.

## 2. Results

### 2.1. ZmbZIP25 Is Silk-Specifically Expressed in Maize

**Figure 1:**
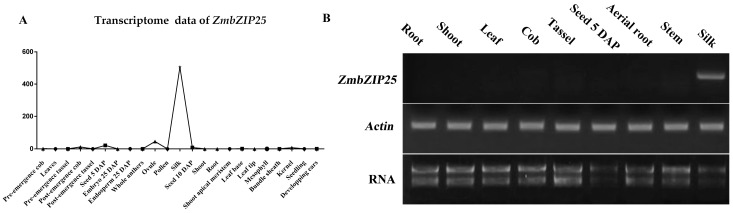
Expression analysis of *ZmbZIP25* in maize. (**A**) Line chart analysis of *ZmbZIP25* transcriptome data. The *x*-axis represents tissues at various developmental periods. The *y*-axis represents transcriptome data (transcriptome data from the MaizeGDB). (**B**) RT-PCR (reverse transcription-polymerase chain reaction) analysis of the expression of the *ZmbZIP25* gene in different maize tissues. RNA was extracted from tissues as indicated above. *Actin* served as a control. DAP, day after pollination.

*ZmbZIP25* (*Zea mays* bZIP (basic leucine zipper) transcription factor 25, GRMZM2G080731) encodes a polypeptide of 346 amino acid residues. ZmbZIP25 belongs to the D group of the bZIP transcription factor family according to its phylogenetic relationship with *Arabidopsis* and rice proteins [17]. Transcriptome sequencing data from MaizeGDB (28/8/2017, http://www.maizegdb.org) indicate that ZmbZIP25 has high-level expression in silks and little expression in other tissues (Figure 1A). To confirm the expression pattern of *ZmbZIP25*, we performed an RT-PCR (reverse transcription-polymerase chain reaction) analysis. The results showed that the *ZmbZIP25* transcript was present in silk but not in other organs, including root, shoot, leaf, cob, tassel, seed 5 DAP (day after pollination), aerial root, and stem (Figure 1B). 

**Figure 2:**
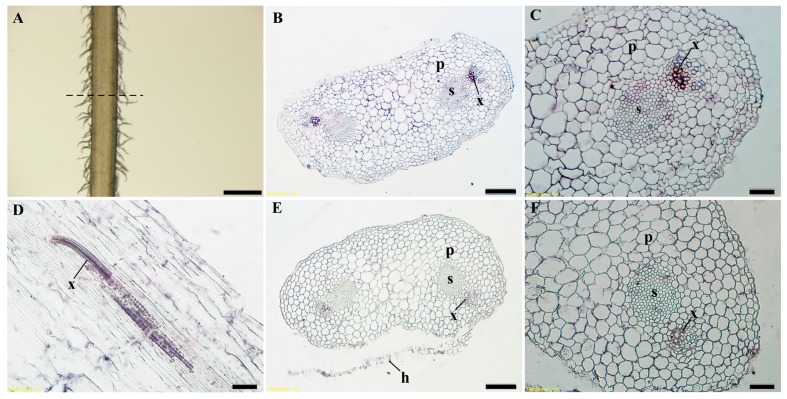
Spatial localization of ZmbZIP25 transcripts in silk. In situ RNA hybridization was performed on silks collected on the day of emergence. (**A**) Fresh maize silk under a stereo microscope, with the dotted line representing the axis of cross sections. Cross section of a silk strand with antisense probes for *ZmbZIP25* (**B**) and enlarged detail (**C**). (**D**) A Sample derived from the longitudinal section of maize silk with antisense probes for *ZmbZIP25*. (**E**) Negative control with sense probes for *ZmbZIP25* and enlarged detail (**F**). Abbreviations: p, parenchyma cell; s, sheath cell; x, xylem; h, hair. Bars = 500 μm in (**A**), 50 μm in (**B**,**E**), and 20 μm in (**C**,**D**,**F**).

To better understand the expression position of ZmbZIP25 in silks, we performed an in situ hybridization analysis using maize silks during flower development. The hybridization results of both cross sections and longitudinal sections showed that ZmbZIP25 was exclusively expressed in the xylem of dumbbell-like maize silk cells (Figure 2).

The protein structure of *ZmbZIP25* was predicted to contain a bZIP domain and a DOG1 (delay of germination 1) domain. DOG1 accumulates during seed maturation and participates in the seed dormancy mechanism of *Arabidopsis*. In situ hybridization assays have indicated that DOG1 is mainly expressed in the vascular tissues of the embryo [18]. ZmbZIP25 was expressed in the vascular tissues of maize silk, which is consistent with the location of DOG1, but was not expressed in maize seed. This result implies that ZmbZIP25 may not participate in seed dormancy. The group D genes of bZIP participate in two different processes: defense against pathogens and development. TGA (TGACG motif-binding factor) transcription factors bind to the as-1 cis-element present in the promoters of pathogenesis-related (PR) genes and induce the expression of PR genes in response to pathogen attack [17]. Maize silks are vulnerable to fungal invasion and insect feeding. The expression of genes in silk is responsible for resisting fungal invasion and insect feeding during pollination and fertilization in maize. ZmbZIP25 may be involved in regulating PR genes.

### 2.2. Analysis of Cis-Regulatory Elements in ZmbZIP25 Upstream Region

To characterize the regulatory mechanisms controlling the transcription of *ZmbZIP25*, we analyzed the upstream region of *ZmbZIP25*. The upstream sequences of *ZmbZIP25* were accessed in Phytozome v12 (https://phytozome.jgi.doe.gov/pz/portal.html#). Surprisingly, a series of Ns was present in the upstream sequences (−443 to −344; predicted transcription start site (TSS) was +1) of the putative *ZmbZIP25* genomic sequence at the website (Figure S1). In addition, multiple occurrences of these upstream series of Ns were found when BLAST searches were performed. The sequence information available online was that of the genetic background of the B73 inbred line. Considering possible errors during sequence splicing, we found the upstream sequences of *ZmbZIP25* from the Mo17 inbred line (from unpublished data), which appears correct and lacks the “Ns” of uncertain nucleic acids. The 5′ upstream region of *ZmbZIP25* was thus cloned from the Mo17 inbred line. 

**Figure 3:**
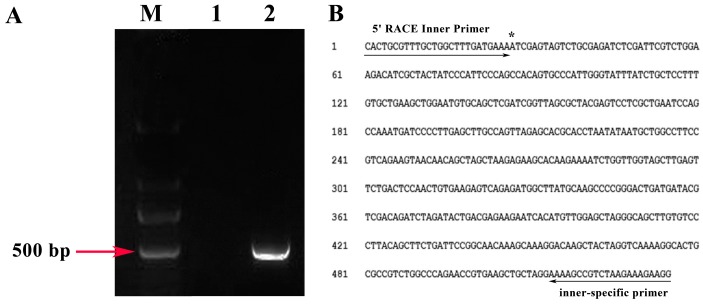
Identification of *ZmbZIP25* transcription start site by 5′ RACE assay. (**A**) 5′ RACE ((rapid amplification of cDNA ends) PCR results. M, DNA marker DL2000 Plus. 1, outer 5′ RACE PCR products. 2, inner 5′ RACE PCR products. (**B**) Sequence alignment result. The arrows represent the primers. The asterisk “*” represents the transcription start site.

A 5′ RACE (rapid amplification of cDNA ends) assay was performed to identify the TSS of the *ZmbZIP25* gene. Amplified fragments (approximately 500 bp in length) were sequenced to determine the 5′ ends of the products. Sequence analyses showed that the *ZmbZIP25* gene contains only one TSS. An adenine residue flanked by cytosine and thymine bases and located 555 bp upstream of the translation start codon initiates gene transcription (Figure 3). This result was consistent with the presence of an adenine in the TSS that is flanked by pyrimidine bases in most plant genes [19]. By comparing cDNA sequences with the genomic sequences of *ZmbZIP25*, we identified two introns in the region between the TSS and the start codon (+67/+160 and +285/+444; the TSS A was designated +1). It is possible that 5′ untranslated regions (5′ UTRs) play roles in regulating gene expression, including regulating mRNA stability and translation efficiency [20]. The 2600 bp fragment from −2083 to +517 that contains upstream sequences and a large part of the 5′ UTR was analyzed. 

**Figure 4:**
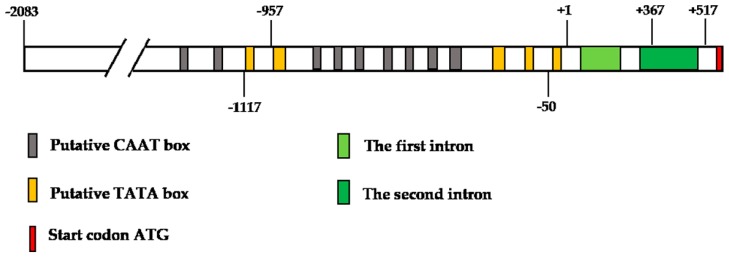
The schematic representation of the 5′-flanking region of *ZmbZIP25*. The transcription start site identified by 5′ RACE was denoted +1. Putative TATA and CAAT boxes in the promoter region are denoted by gray and yellow blocks, respectively. The start codon is denoted by a red block. The first intron and the second intron are denoted by light-green and green blocks, respectively.

Nine putative TATA boxes (core element of promoters, at −1215, −1150, −875, −840, −822, −781, −769, −725, and −552) and five CAAT boxes (proximal element of promoters, at −1113, −941, −452, −285, and −42) were identified in this 2600 bp fragment using the plant cis-acting regulatory element database (http://bioinformatics.psb.ugent.be/webtools/plantcare/html/) (Figure 4 and Figure S2). Interestingly, the putative TATA box was not within 50 bp upstream of the TSS. Nevertheless, two YTCANTYY initiator elements were found in the *ZmbZIP25* 5′-flanking sequence. In addition, some motifs found in the *ZmbZIP25* 5′-flanking sequence, such as the AGCCGCC motif and the GCC-box (http://bioinfo.cau.edu.cn/ProFITS/), are involved in the promoters of many pathogen-responsive genes, which implies that *ZmbZIP25* might be involved in pathogen resistance in maize silk. 

### 2.3. Stigmatic Papillae-Specific Transcription in Arabidopsis and the Impact of the Second Intron of ZmbZIP25

**Figure 5:**
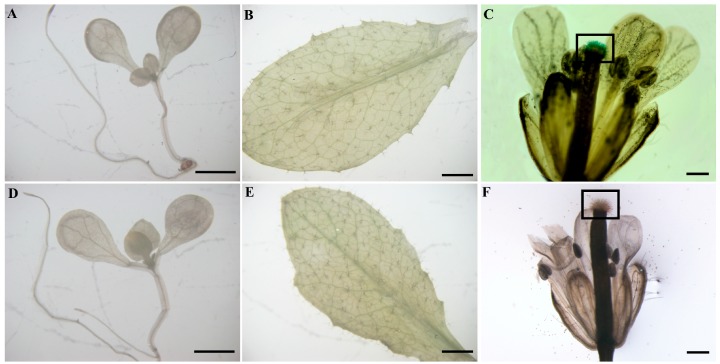
Histochemical analysis of β-glucuronidase (GUS) activity in *Arabidopsis*. Histochemical analysis of GUS activity in seedling (**A**), rosette leaf (**B**), and flower (**C**) of T_2_ transgenic *Arabidopsis* harboring p2450; histochemical analysis of GUS activity in seedling (**D**), rosette leaf (**E**), and flower (**F**) of T_2_ transgenic *Arabidopsis* harboring p2600. The stigmatic papillae are boxed in (**C**,**F**). At least six independent transformants were examined for each construct. Bars = 500 μm in (**C**,**F**), and 2 mm in (**A**,**B**,**D**,**E**).

To characterize the 5′-flanking region of *ZmbZIP25* in plants, we analyzed a 2600 bp fragment ranging from −2083 to +517 and a 2450 bp fragment ranging from −2083 to +367. Heterologous transgenic *Arabidopsis* plants were generated. The plasmids p2600 and p2450 were each transformed into *Arabidopsis* via the *Agrobacterium*-mediated floral dip method. Histochemical analysis of T_2_ generation *Arabidopsis* plants showed that blue staining occurred specifically in the papillae of *Arabidopsis* stigmas (Figure 5C) but not in seedling (Figure 5A) or rosette leaf (Figure 5B) when harboring p2450. Surprisingly, no staining was detected in *Arabidopsis* seedling, rosette leaf, or flower when harboring p2600 (Figure 5D–F). These results indicated that the *GUS* gene was not expressed from p2600. In addition, the 2450 bp fragment (−2083/+367) of the *ZmbZIP25* 5′-flanking region exhibited promoter activity and could specifically drive reporter gene expression. 

**Figure 6:**
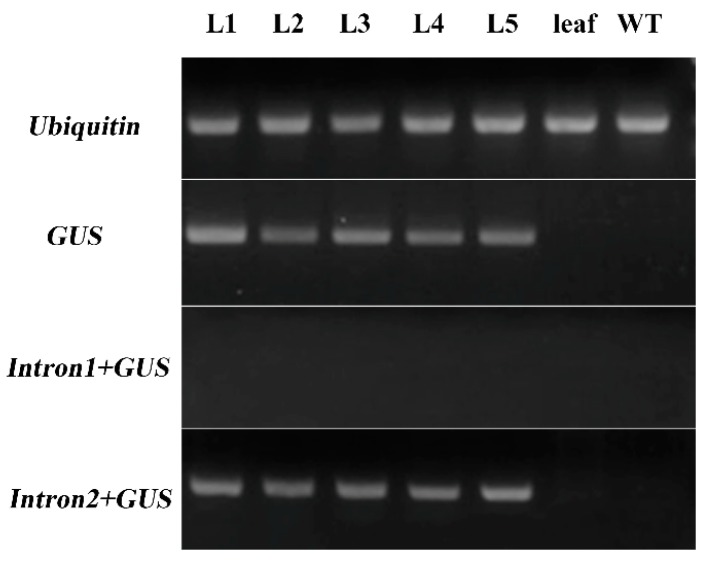
RT-PCR analysis of *GUS* gene in *Arabidopsis*. L1–L5 were cDNA templates from flowers of five p2600 transgenic *Arabidopsis* lines. The two negative control samples were cDNA templates from leaves of p2600 transgenic *Arabidopsis* and non-transgenic *Arabidopsis* flowers. *Intron1+GUS*, the first intron with the *GUS* sequence with the forward primer in the first intron region and the reverse primer in the *GUS* region. *Intron2+GUS*, the second intron with the *GUS* sequence with the forward primer in the second intron region and the reverse primer in the *GUS* region. *Ubiquitin* was used as a control.

The 2450 bp fragment lacked 151 bp at its 3′ end that was present in the 2600 bp fragment. This 151 bp sequence contained a part of the second intron and a part of the 5′ UTR (Figure 4 and Figure S2). Gene expression is influenced by transcriptional regulation, post-transcriptional regulation, and translational regulation. Since GUS was not detected in p2600 transgenic *Arabidopsis*, the level of the *GUS* mRNA was tested. RT-PCRs showed that *GUS* gene transcripts were present in p2600 transgenic *Arabidopsis* flowers but not in the corresponding leaf (Figure 6). In other words, *GUS* gene transcription into mRNA could be specifically driven by the 2600 bp fragment of *ZmbZIP25*, but this mRNA was not translated correctly. The second intron might not be correctly spliced during the formation of mature mRNA. RT-PCR was performed to test this assumption. The forward primer was in the intronic region, and the reverse primer was in the *GUS* region. The first intron with a *GUS* sequence (*Intron1+GUS*) could not be amplified, while the second intron with a *GUS* sequence (*Intron2+GUS*) was successfully amplified (Figure 6). The RT-PCR products were sequenced and confirmed. These results verified that the second intron was still present in the transcripts, which indicated that the inability to drive the expression of the *GUS* gene with the 2600 bp fragment of *ZmbZIP25* in transgenic lines might be due to aberrant pre-mRNA splicing.

### 2.4. Silk-Specific Transcription in Maize

**Figure 7:**
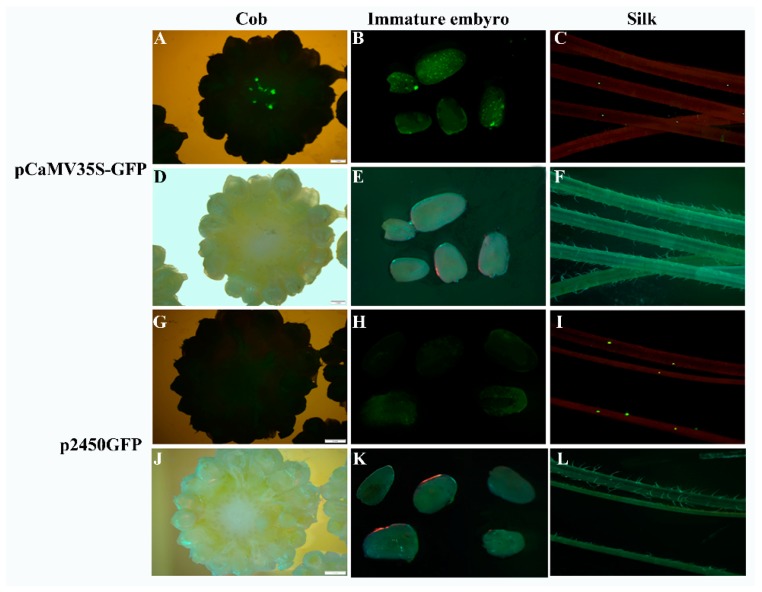
Silk-specific expression driven by the *ZmbZIP25* promoter in maize via microprojectile bombardment. Green fluorescence of GFP (green fluorescent protein) expression detected in cob (**A**), immature embryos (**B**), and silks (**C**) bombarded with the vector pCaMV35S-GFP. The corresponding images (**D**–**F**) were photographed under light microscopy. Green fluorescence of GFP expression detected in cob (**G**), immature embryos (**H**), and silks (**I**) bombarded with the vector p2450GFP. The corresponding images (**J**–**L**) were photographed under light microscopy. Bars = 2 mm in (**A**,**D**,**G**,**J**), and 1 mm in (**B**,**C**,**E**,**F**,**H**,**I**,**K**,**L**).

To examine the silk-specific expression driven by the *ZmbZIP25* promoter in maize, we performed transient expression assays via microprojectile bombardment and *Agrobacterium*-mediated transformation. After bombarding vectors p2600GFP and p2450GFP into various tissues of maize, we detected GFP (green fluorescent protein) expression. No fluorescence was detected in maize cob, silks, or immature embryos harboring p2600GFP. Maize harboring p2450GFP exhibited green fluorescence in its silks but not in its cob or immature embryos (Figure 7G–L). The vector pCaMV35S-GFP was bombarded to serve as a positive control, resulting in green fluorescence in cob, immature embryos, and silks (Figure 7A–F). 

**Figure 8:**
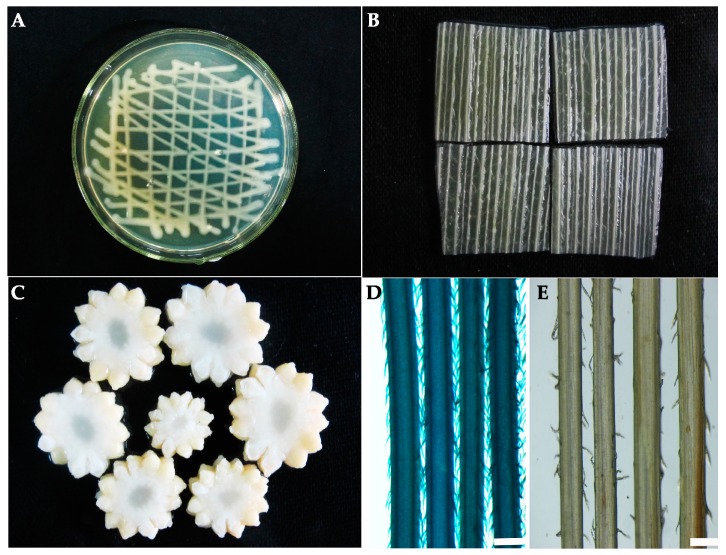
Silk-specific expression driven by the *ZmbZIP25* promoter in maize via *Agrobacterium*-mediated transformation. Histochemical GUS analysis of p2450-transformed maize husk (**B**), cob (**C**), and silks (**D**), and *Agrobacterium* (**A**), which served as a background. (**E**) Histochemical GUS analysis of p2600-transformed maize silks. Bars = 500 µm in (**D**,**E**).

Vectors p2600 and p2450 were also used to probe the expression of the *GUS* gene driven by the different *ZmbZIP25* promoter fragments. *Agrobacterium*-mediated transformation was performed in maize husk, cob, and silk. Histochemical analysis showed that blue staining was not observed in tissues carrying p2600 (Figure 8E). In p2450-transformed tissues, blue staining was observed in maize silk (Figure 8D) but not in maize husk (Figure 8B), or cob (Figure 8C), or *Agrobacterium* (Figure 8A), which served as a background control. 

Transient expression assays in maize consistently showed that the expression of reporter genes was specifically driven by the 2450 bp fragment (−2083/+367) of *ZmbZIP25* and not by the 2600 bp fragment (−2083/+517). The results also verified conclusions drawn from the stable expression assays in *Arabidopsis*.

### 2.5. The −1117 to −957 Region is Responsible for the Silk Specificity of the ZmbZIP25 Promoter

**Figure 9:**
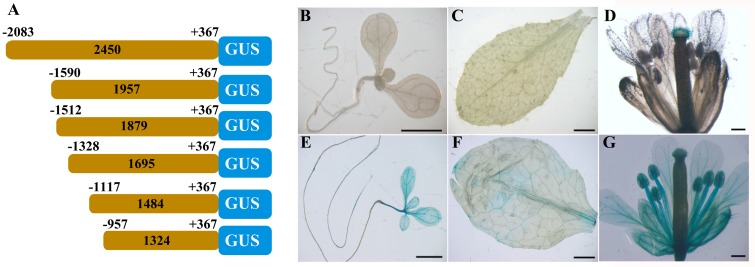
Deletion analysis of *ZmbZIP25* 5′-flanking sequence. (**A**) A schematic map of *ZmbZIP25* promoter deletion constructs fused to GUS reporter gene that were used for loss-of-function analysis. The gray boxes represent various *ZmbZIP25* 5′-flanking sequences. The blue boxes represent *GUS* gene. (**B**–**G**) Histochemical staining of GUS gene driven by truncated *ZmbZIP25* 5′-flanking fragments in *Arabidopsis*. *ZmbZIP25* 5′-flanking fragment (−1590/+367) in *Arabidopsis* seedling (**B**), rosette leaf (**C**), and flower (**D**); *ZmbZIP25* 5′-flanking fragment (−957/+367) in *Arabidopsis* seedling (**E**), rosette leaf (**F**), and flower (**G**). At least six independent transformants were examined for each construct. Bars = 500 μm in (**D**,**G**), and 2 mm in (**B**,**C**,**E**,**F**).

To determine the specific region involved in *ZmbZIP25* gene expression, we generated a series of 5′ deletion fragments (Figure 9A). Five vectors (p1957, p1879, p1695, p1484, and p1324) were constructed and separately transformed into *Arabidopsis thaliana* col-0. Histochemical analysis indicated that the *GUS* gene was specifically expressed in *Arabidopsis* stigmatic papillae harboring p1957 (−1590/+367) (Figure 9B–D) as well as those of the p1879 (−1512/+367), p1695 (−1328/+367), and p1484 (−1117/+367) transformants. These results implied that the truncated sequences ranging from −1117 to +367 retained full function and specificity. When the fragment was truncated to 1324 bp (−957/+367), the histochemical assay showed GUS staining in many tissues, including seedling (Figure 9E), rosette leaf (Figure 9F), and whole floral organs (Figure 9G). GUS activity differed in various tissues when p1324 was harbored; this activity was relatively low in seedling root and rosette leaf but relatively high in seedling leaf and the whole flower (Figure 9E–G). This result illustrated that the GUS activity was certainly driven by the region from −957 to +367 but that this region did not encode specificity. The 1324 bp sequence (−957/+367) was 161 bp shorter than the 1484 bp sequence (−1117/+367). GUS activity was detected in seedling, rosette leaf, and the whole flower when this 161 bp sequence was lacking, suggesting the possible presence of negative regulatory elements in the region from −1117 to −957 that suppress gene expression in tissues other than stigma.

We subsequently analyzed the −1117 to −957 sequence of the *ZmbZIP25* promoter. A Box C element (CTCCCAC) was found in the 161 bp region between −1117 and −957 but not in any other positions in the *ZmbZIP25* promoter (09/08/2017, http://bioinfo.cau.edu.cn/ProFITS/). The Box C element in the pea asparagine synthetase (AS1) promoter is involved in light-induced transcriptional repression [21]. Box C may be the negative regulatory element causing tissue specificity in maize silks of *ZmbZIP25*.

## 3. Discussion

ZmbZIP25 contains a bZIP domain and a DOG1 domain and belongs to the D group of the 11 groups of the maize bZIP proteins according to its phylogenetic relationship [17,22]. Genes in group D might participate in the pathogen response [17]. *TGA6*, which belongs to the TGA family in bZIP group D, is speculated to function in defense against pathogens in *Arabidopsis* [23]. Bioinformatic predictions indicate that some genes in group D that show similar patterns of expression are involved with fungal infections [17]. TGA2 and TGA3 interact with NPR1, bind SA-responsive elements in the *Arabidopsis* pathogenesis-related (PR-1) promoter, and are involved in disease resistance pathways [24]. *ZmbZIP25* may regulate the expression of pathogen resistance genes in maize silk by interacting with other factors. The exact function and regulation of *ZmbZIP25* are being investigated.

bZIP transcription factors may regulate cell-type-specific gene expression in plants. Grp1.8 (glycine-rich protein) exhibited xylem-specific expression in tomato. bZIP transcriptional activator VSF-1 bound a 28 bp element in the *grp1.8* promoter (vs-1) to regulate *grp1.8* expression [25]. Transcriptome data and RT-PCR results showed that *ZmbZIP25* had high-level expression in silks and was barely detected in other tissues (Figure 1). In situ hybridization assays indicated that *ZmbZIP25* was expressed exclusively in the xylem of maize silk cells (Figure 2). Few genes have been reported that are expressed specifically in the xylem of maize silk. Our research on *ZmbZIP25* enriches the study of xylem-specific promoters. 

A 5′ RACE assay was performed to identify the TSS of *ZmbZIP25* (Figure 3). However, no TATA box was present within 50 bp upstream of the TSS. Two YTCANTYY initiator elements were found in the *ZmbZIP25* promoter (http://bioinfo.cau.edu.cn/ProFITS/) (Figure 4 and Figure S2). In some TATA-less promoters, pyrimidine-rich initiator (Inr) elements that overlap the TSS compensate for the lack of a TATA box and direct basal transcription initiation [26,27,28]. The tobacco photosystem I *psaDb* gene promoter responds to light and depends on an *Inr* but not a TATA box [29]. The promoter of *Pib*, a member of the NBS-LRR (nucleotide-binding site leucine-rich repeat) class of plant disease resistance genes, contains six YTCANTYY motifs and makes the expression of *Pib* in darkness specific to root [30,31]. The *ZmbZIP25* promoter is expressed exclusively in silk and may be associated with pathogen resistance based on the YTCANTYY motif. Some pathogen-responsive elements, such as the AGCCGCC motif and the GCC-box, are also present in the *ZmbZIP25* promoter. The precise mechanism by which *ZmbZIP25* participates in pathogen resistance remains unknown, but further research is underway. 

Considering that the stable expression assay in maize plants requires a long period of time and that its low transformation efficiency makes it difficult to realize, the model plant *Arabidopsis* was selected as an alternative system due to its relatively short life cycle and highly efficient transformation [32]. The histochemical analysis of *GUS* genes showed that blue staining occurred in *Arabidopsis* stigma papillae only when harboring p2450 (and not when harboring p2600, Figure 5). These results were also confirmed via transient expression assays in maize (Figure 7 and Figure 8). 

The difference between the 2450 and 2600 bp fragments was a deletion of 151 bp from the 3′ end. This 151 bp region contains part of the second intron and part of the 5′ UTR (Figure 4 and Figure S2). RT-PCRs verified that the second intron was still present in transcripts (Figure 6). The precise removal of pre-mRNA introns is critical for gene expression [33]. The conformation of the pre-mRNA influences the accessibility of splice sites. The number of structural options increases with the molecule’s length. The secondary structures of *GUS* pre-mRNA in the p2600 transgenic line may differ from those in the p2450 transgenic line, affecting pre-mRNA splicing. A splicing error that adds or removes even 1 nt will perturb the open reading frame of an mRNA [34]. Aberrant pre-mRNA splicing of the second intron of p2600 might lead to the inability to express the *GUS* gene in transgenic *Arabidopsis*. 

A gene that displays specific expression may be influenced by its regulatory elements, including negative elements and interacting transcriptional factors. In plants, the sequence CGTG(T/C)G of the *CPD* (constitutive photomorphogenesis and dwarfism gene) promoter was tested as a binding site for transcription repressor BZR1 to regulate brassinosteroid signaling and downstream growth responses [35]. In *Arabidopsis*, FRS7 and FRS12 affect flowering time and growth partly by binding to the promoters and repressing the expression of *GIGANTEA* and *PHYTOCHROME INTERACTING FACTOR 4* as well as several of their downstream signaling targets [36]. A series of deletion analyses showed that a reporter gene driven by the truncated 1324 bp region (−957/+367) in *ZmbZIP25* was expressed not only in the stigmatic papillae but also in other tissues (Figure 9). This result implied the possible presence of negative cis-elements in the region of *ZmbZIP25* from −1117 to −957 that can suppress gene expression in tissues other than silk. A Box C element (CTCCCAC) was found only within the 161 bp region from −1117 to −957 (09/08/2017, http://bioinfo.cau.edu.cn/ProFITS/), which was identified as a cis-element in the pea asparagine synthetase (AS1) promoter that participated in light-induced transcriptional repression [21]. We speculate that Box C tends to be a negative regulatory element in *ZmbZIP25* and functions in a tissue-specific manner in maize silks. To confirm the negative regulatory cis-element sequences in the region from −1117 to −957 of *ZmbZIP25*, further studies are required, such as those involving the creation of point mutations in the promoter and the examination of DNA-protein binding and protein-protein interactions. 

In conclusion, our findings suggest that the region from −1117 to −957 of the *ZmbZIP25* 5′-flanking sequence is necessary for silk-specific gene expression. The second intron of *ZmbZIP25*, involved in aberrant pre-mRNA splicing, may provide insight into tissue-specific promoter research. The cis-elements and mechanism of *ZmbZIP25* silk-specific expression should be further explored. Moreover, the *ZmbZIP25* 5′-flanking sequence, as a strong silk-specific promoter, has potential agricultural application value in areas such as female sterility.

## 4. Materials and Methods

### 4.1. RT-PCR Analysis

Total RNA was isolated from various tissues of maize (*Zea mays* L. cv Mo17) using an RNAprep Pure Plant Kit (Tiangen, Dalian, China) according to the manufacturer’s protocol. The first-strand cDNA was synthesized with AMV (avian myeloblastosis virus) reverse transcriptase (Promega, Madison, WI, USA). The *ZmbZIP25* transcription products were amplified using the specific primers RT-ZIP-F (5′-CGACCAGCAGCCAAACTCTA-3′) and RT-ZIP-R (5′-AATCCGCCCAGCCACATAAA-3′). The maize actin gene was used as a reference control. This amplification experiment was repeated three times. 

A similar procedure was performed in *Arabidopsis*. *GUS* transcription products were amplified using the specific primers GUS-F (5′-CGACTGGGCAGATGAACATG-3′) and GUS-R (5′-TACTCCACATCACCACGCTT-3′). The transcription product containing the first intron and *GUS* (*Intron1+GUS*) was amplified using the specific primers F1 (5′-TGCTCCAGGCAACCTTGTTT-3′) and R1 (5′-TCCACAGTTTTCGCGATCCA-3′). The transcription product containing the second intron and *GUS* (*Intron2+GUS*) was amplified using the specific primers F2 (5′-CCGGTGAGCCGATGATTTCT-3′) and R2 (5′-CCTGCCCAACCTTTCGGTAT-3′). The *Arabidopsis* ubiquitin gene was used as a reference control. 

### 4.2. In Situ RNA Hybridization 

Fresh silks were collected from maize plants on the day of emergence, cut into 2–3 mm lengths, and then immediately fixed in FAA solution (50% ethanol, 5% formaldehyde, and 5% acetic acid). The gene-specific region of *ZmbZIP25* (+631 to +1035, counted from the ATG start codon) was amplified as a template for synthesizing sense and antisense RNA probes. The probes were labeled with digoxigenin using a DIG Northern Starter Kit (Roche Diagnostics, Mannheim, Germany). The silks were dehydrated, embedded in paraffin (Sigma-Aldrich, Saint Louis, MO, USA), sliced to an 8 μm thickness, pretreated, hybridized, washed, and detected as previously described [37].

### 4.3. Identification of ZmbZIP25 Gene Transcription Start Site

To identify the TSS of the *ZmbZIP25* gene, 5′ RACE was performed using a FirstChoice^®^ RLM-RACE Kit (Ambion, Vilnius, Lithuania). Total RNA was treated with calf intestinal phosphatase (CIP) and then decapped with tobacco acid pyrophosphatase (TAP). A 45 base RNA adapter oligonucleotide was ligated to the RNA population using T4 RNA ligase. A random-primed reverse transcription reaction and nested PCR were then used to amplify the 5′ end of a specific transcript. Two gene-specific antisense primers (outer-specific primer: 5′-AGTAGAGTTTGGCTGCTGGT-3′ and inner-specific primer: 5′-CCTTCTTTCTTAGACGGCTTTT-3′) were designed to amplify with the sense primers provided with the kit. The amplified PCR products were sequenced.

### 4.4. Construction of Expression Vectors

The 5′-flanking sequences of *ZmbZIP25* were amplified by PCR, and a 2600 bp fragment (−2083/+517) and a 2450 bp fragment (−2083/+367) were used to construct vectors. We used a pCaMV35S-GFP backbone and the above fragments to construct p2600GFP and p2450GFP, respectively, which no longer had the CaMV35S promoter to drive the expression of the *GFP* reporter gene. pCaMV35S-GFP, which served as a control, was modified from vector pUC-SPYCE by replacing YFP^C^ with GFP [38].

Seven different promoter fragments (−2083/+517, −2083/+367, −1590/+367, −1512/+367, −1328/+367, −1117/+367, and −957/+367) were cloned. These fragments were inserted into the binary vector pCAMBIA1391 at its *Eco*RI and *Spe*I sites to drive *GUS* gene expression. The created vectors were named p2600, p2450, p1957, p1879, p1695, p1484, and p1324, respectively.

### 4.5. Microprojectile Bombardment and Transient Expression Assay in Maize

Maize plants were grown in soil in a controlled-environment greenhouse (30 °C, 14 h light/20 °C, 10 h dark). The cobs and silks were obtained separately from fresh non-pollinated maize ears. Cobs were cut into 5-mm-thick cross sections. Silks were cut into approximately 2.5 cm lengths. The immature embryos were peeled 15 d after pollination. All the tissues were pretreated with hypertonic medium (0.4 M mannitol in NB Basal medium) for 4 h before bombardment. A biolistic PDS-1000/He system (Bio-Rad, Hercules, CA, USA) was used to bombard the plasmid DNA p2600GFP, p2450GFP, and pCaMV35S-GFP, carried by gold particles, under 1300 psi of helium. After bombardment, the tissues were maintained in the hypertonic medium overnight. GFP fluorescence was observed under an Olympus fluorescence microscope (SZX-16, Tokyo, Japan).

### 4.6. Agrobacterium-Mediated Transformation and Transient Expression Analysis

*Agrobacterium*-mediated transformation was performed according to the method of Frame et al. [39] with some modifications. Various tissues of maize, including husk, cob, and silk, were freshly harvested from greenhouse-grown maize plants and infected with an *Agrobacterium* strain GV3101 (harboring plasmid p2600 or p2450) suspension (OD_600_ = 0.3–0.4) for 30 min. After infection, all the tissues were dried on filter paper, transferred to co-cultivation medium, and then incubated in the dark at 20 °C for 2 days. Before they were stained, the tissues were washed with ddH_2_O to remove the *Agrobacterium* cells from the tissue surfaces. Histochemical analysis was performed to test the GUS activity.

### 4.7. Arabidopsis thaliana Transformation and Selection

*Arabidopsis thaliana* Columbia-0 was transformed with *Agrobacterium tumefaciens* strain GV3101 cells harboring a plasmid (p2600, p2450, p1957, p1879, p1695, p1484, or p1324) using the floral dip method [40,41]. Transformed seeds were germinated on MS medium (Murashige and Skoog medium) containing 50 mg/L hygromycin for selection. Resistant transgenic *Arabidopsis* plants were confirmed by PCR with the primers bZIP25-F (CGACCAGCAGCCAAACTCTA) and bZIP25-R (AATCCGCCCAGCCACATAAA). More than five independent lines of the T_2_ generation were used for further analysis.

### 4.8. GUS Activity Assays

Histochemical assays for GUS activity were performed as described by Lang et al. [42], with modification. The GUS reaction buffer contained 100 mM sodium phosphate buffer (pH 7.0) with 1 mg/mL X-Gluc (5-bromo-4-chloro-3-indolyl-β-d-glucuronide) as the substrate with 5 mM K_3_Fe(CN)_6_, 5 mM K_4_Fe(CN)_6_, 0.5 mM EDTA (Ethylenediaminetetraacetic acid), 0.1% Triton X-100, and 20% methanol. Samples were vacuum-filtered and then incubated in GUS reaction buffer at 37 °C overnight in the dark. Then, 70% ethanol was used for decoloration. The samples were photographed under a stereo microscope (Olympus SEX16, Tokyo, Japan).

## Supplementary Materials

The following are available online at http://www.mdpi.com/1422-0067/19/3/822/s1.

## Abbreviations

bZIP	basic leucine zipper
DOG1	delay of germination 1
PR	pathogenesis-related
5′-UTR	5′-untranslated region
