# Supplementary material for: Functional Analysis of Maize Silk-Specific ZmbZIP25 Promoter
Source: Int J Mol Sci. 2018 Mar 12;19(3):822. doi: 10.3390/ijms19030822 (PMC5877683; doi:10.3390/ijms19030822)
Supplement: Supplementary file 1 [file ijms-19-00822-s001.pdf]

Functional Analysis of Maize Silk-Specific *ZmbZIP25* Promoter

Wanying Li, Dan Yu, Jingjuan Yu, Dengyun Zhu and Qian Zhao *

State Key Laboratory of Agrobiotechnology, College of Biological Sciences, China Agricultural University, No. 2 Yuanmingyuan West Road, Haidian District, Beijing 100193, China; liwanyingcau@foxmail.com (W.L.); danyupw155@yeah.net (D.Y.); yujj@cau.edu.cn (J.Y.); zhudy@cau.edu.cn (D.Z.)

* Correspondence: zhaoqian@cau.edu.cn; Tel.: +86-10-6273-3330

>GRMZM2G080731 | 8:119662868..119665630 forward

CCGTCCGAGCCCTCGCGCGGACCGTCCGGCCTCAGGCGCGGACCGTCCGGTCGGTAAGAAACCGAAAAACCCGAAGGTGACGGGTTCGGAGAAATGAATT
TTAGCGGCCTCGCGGACCGTCCGGGCCAGGCGGGCGGACCGTCCGCGACTGGCTCTGTCTGACATCTGACGTCGCATTAAATGCAATATAGCCGTTGATA
TAGCCGTTACTGCTGACCGTTGCATCTTCAGCTGTTGATCTACAGGGGCGGACCGTCCGCACCAGGAGGGCGGACCGTCCGCGCTCAGCAGAAAGGCCCA
ACGGCTAGGAAGTGGTTGGTGGCTATAAATACAACCCCAACCACCTCCATTCAATCTATCCAAGCATTCCAACCTTCAACATTCAATACAAGAGCTAGCA
ATCCATTCCAAGACACATTCAAAGCCTCCATCTCTCCAAGTTTCGAAATTGAGATAAGAGATCATTAGTGATTAGTGGCTTTGTTTTTAAGTGATCCGTG
TGTCATTTGTCGCTCTTGTTGCTGGCAACGAAATTCAAGCTGAATCATAAGAAACCANNNNNNNNNNNNNNNNNNNNNNNNNNNNNNNNNNNN
NNNNNNNNNNNNNNNNNNNNNNNNNNNNNNNNNNNNNNNNNNNNNNNNNNNNNNTCGTGGATAGATTTCTCGAGATTCTCAGCTTGCATCCGATCCA
GCAAGCTCTCAGTTCCGTTTAGCCCCCCACTAATGACTAAAAGTAGACTGAAAAGTTGAGACGCGCGCACATGCAGCTACGCCGCTACGCTATGGCTGGT
TCACCCACCGGAGCCTGCAACGCGCTCCCTGTGGAGAGAGAAAAGAAGCTCTGCCACCCGTCCACACATTCCTGTGATAGATCGCATTCATGCAGCTGGC
TACTGAATGCGCGTTGTGCTGCGTAGGACACGTATGGCGAGCTAGCCTACAGATAAGCCTGGGGAATGGTTTCATATGCATCCGGCTCGCGCAGGTTTCT
GGCATCGAGCAGTCTGCGAGATCTCGATTCGTCTGGAAGACATCGCTACTACCCCAGCCACAGGTATGCTGTGCTGCTAAGTTGCTCCAGGCAACCTTGT

Figure S1. **The region with a green background represents the 5' UTR of *ZmbZIP25* in Phytozome.**

-2083 TGCTGAGGAGGAGGAAGTTCTCGGGCTTGAGGTCGCGGTGGATGACGCCCATGGAGTGGC
-2023 AGGTGTGCACGATCTCGACGATGGTGCGGAGCAGCGAGGCGGCGGCGCTCGGTGTAGT
-1963 GGCCCCTGGCGATGATGCGGTCGAAGAGCTCGCCGCCGGCGCAGAGCTCCATGACGAGGT
-1903 GCACGGAGCTTTTGTCCTCGTACGCGCCCTTGAGCTCCACCACGCCCGGCTGGCCGGACA
-1843 GGTGGTACATGATCTGTACCTCGCGCCGCACGTCCTCCACGTCCTCCTTGGTGGACAGCT
-1783 TCCGCTTGCTGATGGTCTTGCACGCGAACCTGTGCCCCGTGGCCTTGTGCGTGCACAGCG
-1723 ACGTGACGCCGAACTGCCCGCGCCCGAGCTCCTTGCCCACGGCGTAGGTGGCCCTCACGT
-1663 CCTCCATGGGCCGGCCCAGCACGGGGCCGATGGGGGCCGGGGGCCTGGCGGGCGCGGGCG
-1603 CCGCGGTCGGCGACACCGAGGCAGGCGAAGCCACGGGGCGCGACGAGCCGGAGGGGTCCG
-1543 GCGGCTGCGGCGGCGCGGGCTCCGCGGACGACGACGACCCCGGGCAGCAGTTGCCCATGT
-1483 CGGCGACGGCGAGGGGGGCGACGGAGACGCGGGGGCCTCGCGCGCCGGGGACGCGGACGC
-1423 GCAGGCGCGGGCGCAGCGAACCGGGGAGGTGTCCGGGCGAGCGGTGGGGCGCGCGGGGGA
-1363 ACCTTTCTCGGACGGGTGGGAGCGCTGGCCATGAAAGGGAAGGCTGGGAGGAAATGGCGG
-1303 GAACGTAACGGCTGCTTGCTTTTGCCTTCTGTTGCCTTGTTTATGCTCAACAGAGAAAAA
-1243 CAACAGAAGCTTGCGTCCAACTTCTCTATTTTAGCACCAGGCCCATAAATCATAGACACA
-1183 GATGGTCTCATAAGTCATAAACAGAGGATCACATAATAGCATTTAGCCCACATTAAAAGG
-1117
-1123 GCTAAAATAGCAAATTTTTCCCTGCGTCCGTGCACCCACGCCGAGCTTGCCGCTGCGACC
-1063 GTGGCCGAGCAGATCACGCACACGACATGCATTGCTTTGTTCGTCTTCAAAACAAACCCA
-957
-1003 AAAAAAATGGTTTTGCGGTTTTGCCCGACCAGCCTGGAGCACGGTGGTGCTCAGCCAGAC
-943 AGCCAATAGGACATTCTTGATTCTTGATGTTGTATTGTATTTGTTGAGTTTGTGATCTAA
-883 ATTTTGTTTATAGGACATATTAGCAAGTAAAGCGGAGGCTTGTTATAGGTTTGGGCCGGA
-823 GTATATTGCTGACTCTAATTAGGGTTTCTGTTGCGTCATAATTTTTACTCCTCTTATAAG
-763 CCGTCGTACAAAGACGGCGTTATGTAAACATTTCTACATATAGTGAAGGTTTTCGTAATT
-703 TTTCTCTTTGCATTAAGGACCTGTTTGGTTAGAGGTCTCTCAGGCACTTGCCTGCCAAGT
-643 AAGGAATCCTAGCCCATGTATTCAAAATCTAACGCCTGAGATCTGTGCAACTTTGGCCGG
-583 CTCCAAACTAAATAAGCCTTAAAAGAATTTCTATGTAAATCTATGTATGGTGCTCTGTGG
-523 TTTGTTGTTTGATCTTCCCTGTTTTGTCTGCTAACGGTATCCATCCGTCCATTGATGCTC
-463 CGGCCCTTTCCCAATGTCCTGTGCTGGAATCTTGATAGCTTGGGGCAGTTGAGTTTGGCG
-403 TGTGAAGTGGACGTGGACTTGGCGTTGGATAGATTCTGCAAGCTTTCGTGGATAGATTTC
-343 TCGAGATTCTGAGCTTGCATCCGATCCAGCAAGCTCTCAGTTCCGTTTAGGCCCCCCACA
-283 AATGACTAAAAGTAGACTGAAAAGTTGAGACGCACGCACATGCAGCTACGCCGCTACGCT
-223 ATGGCTGGTTCACCCACCGGAGCCTGCAACGCGCTCCCTGTGGAGAGAGAAAAGAAGCTC
-163 TGCCACCCGTCCACACATTCCTGTGATAGATCGCATTCATGCAGCTGGCTACTGAATGCG
-103 CGTTGTGCTGCGTAGGACACGTATGGCAAGCCTGGGGAATGGTTTCATGTGCTCTGTTCT
+1
-43 ACAATGTACATACATATGCATCCGGCTCGCGCAGGGGCGTGGCATCGAGTAGTCTGCGAG
+18 ATCTCGATTCGTCTGGAAGACATCGCTACTATCCCATTCCCAGCCACAGGTATGCTGTGC
+78 TGCTAAATTGCTCCAGGCAACCTTGTTTTGAACTCGTTTTTGCTGAACAACTAACTAGCT
+138 GCTTCGATTTTTTGACTGTGCAGTGCCCATTGGGTATTTATCTGCTCCTTTGTGCTGAAG
+198 CTGGAATGTGCAGCTCGATCGGTTAGCGCTACGAGTCCTCGCTGAATCCAGCCAAATGAT
+258 CCCCTTGAGCTTGCCAGTTAGAGCACGGTGAGCCTATTGTACTCTGATCATATCCGGTGA
+367
+318 GCCGATGATTTCTTAGAGCAAGATTAAAATAGCCTCACCCTCACAGCAGGCTTTTATATG
+378 GTTCAGATCTGCAGATCTGTCTGTCTCCCATTCATTCTTCTTTGCTTTTTTTTTTTTGGA
+438 AGCGCAGCACCTAATATAATGCTGGCCTTCCGTCAGAAGTAACAACAGCTAGCTAAGAGA
+517
+498 AGCACAAGAAAATCTGGTTGGTAGCTTGAGTTCTGACTCCAACTGTGAAGAGTCAGAGATG

Figure S2. Sequences analysis of the 5'-flanking region of *ZmbZIP25*. The transcription start site identified by 5' RACE is represented by a bold red *"A"* at +1. The translation initiation codon is boxed in black. Two intron sequences are denoted with a gray background. The first intron is the region from +67 to +160. The second intron is the region from +285 to +444. Putative TATA and CAAT boxes in the promoter region are in blue letters.
